# MicroRNA regulation in blood cells of renal transplanted patients with interstitial fibrosis/tubular atrophy and antibody-mediated rejection

**DOI:** 10.1371/journal.pone.0201925

**Published:** 2018-08-13

**Authors:** Mareen Matz, Frederik Heinrich, Christine Lorkowski, Kaiyin Wu, Jens Klotsche, Qiang Zhang, Nils Lachmann, Pawel Durek, Klemens Budde, Mir-Farzin Mashreghi

**Affiliations:** 1 Department of Nephrology, Charité University Medicine Berlin, Berlin, Germany; 2 Deutsches Rheuma-Forschungszentrum Berlin, a Leibniz Institute (DRFZ), Berlin, Germany; 3 Department of Pathology, Charité University Medicine Berlin, Berlin, Germany; 4 Center for Tumor Medicine, HLA Laboratory, Charité University Medicine Berlin, Berlin, Germany; Institut de Pharmacologie Moleculaire et Cellulaire, FRANCE

## Abstract

Interstitial fibrosis/tubular atrophy (IFTA) is associated with reduced allograft survival, whereas antibody-mediated rejection (ABMR) is the major cause for renal allograft failure. To identify specific microRNAs and their regulation involved in these processes, total RNA from blood cells of 16 kidney transplanted (KTx) patients with ABMR, stable graft function (SGF) and with T-cell mediated rejection (TCMR) was isolated. MicroRNA expression was determined by high-throughput sequencing. Differentially expressed candidate microRNAs were analyzed with RT-PCR in patients with SGF (n = 53), urinary tract infection (UTI) (n = 17), borderline rejection (BL) (n = 19), TCMR (n = 40), ABMR (n = 22) and IFTA (n = 30). From the 301 detected microRNAs, 64 were significantly regulated between the three cohorts. Selected candidate microRNAs miR-223-3p, miR-424-3p and miR-145-5p distinguished TCMR and ABMR from SGF, but not from other pathologies. Most importantly, miR-145-5p expression in IFTA patients was significantly downregulated and displayed a high diagnostic accuracy compared to SGF alone (AUC = 0.891) and compared to SGF, UTI, BL, TCMR and ABMR patients combined (AUC = 0.835), which was verified by cross-validation. The identification of miR-145-5p as IFTA specific marker in blood constitutes the basis for evaluating this potentially diagnostic microRNA as biomarker in studies including high numbers of patients and different pathologies and also the further analysis of fibrosis causing etiologies after kidney transplantation.

## Introduction

MicroRNAs are small, non-coding RNAs that inhibit translation of their complementary target mRNA thereby controlling gene expression. Many distinct mRNAs can be silenced by a single microRNA and a single transcript can be regulated by several microRNAs. Predictably 30–80% of human genes might be regulated substantially by microRNAs, including genes involved in the underlying causes of variable diseases and disorders. The regulation of microRNAs has been extensively studied in the context of renal disease, including in cancer [1, 2] and fibrosis [3, 4]. After kidney transplantation (KTx), microRNAs have been observed to be regulated in antibody-mediated rejection (ABMR) [5, 6], interstitial fibrosis/tubular atrophy (IFTA) [7, 8] and acute rejection [9–13] proposing their potential value as non-invasive biomarker. The main focus of biomarker research and also functional and regulatory studies in the microRNA field is currently shifting to the areas of ABMR and IFTA, especially when interstitial inflammation is present. These pathologies after kidney transplantation are deleterious to the graft, limit long-term outcomes and are the main risk factors for graft loss [14, 15]. The diagnosis relies on conventional invasive grading and screening methods and is therefore limited, whereas effective treatment strategies are lacking. Those challenges originate from the still insufficient knowledge about the specific triggering mechanisms causing chronic allograft failure. The search for markers like microRNAs that are regulated during pathological processes and subsequent in-depth functional investigations will allow the translation of the findings into personalized management of KTx recipients by the development and the standardization of diagnostic, monitoring and therapeutic strategies for ABMR and IFTA.

Based on high-throughput sequencing experiments with blood cells of renal transplanted patients with ABMR, T-cell mediated rejection (TCMR) and stable graft function (SGF), specific microRNA candidates were validated in a large patient cohort with different pathologies. The candidates did not prove to be highly specific for ABMR, but the candidate miR-145-5p was significantly down-regulated in patients with IFTA compared to patients with ABMR, SGF, urinary tract infection (UTI), borderline rejection (BL) and TCMR. We here present first indications that miR-145-5p might have diagnostic properties for IFTA after KTx and hypothesize that this marker might also be involved in the molecular mechanisms leading to fibrosis of the graft.

## Materials and methods

### Patients and sample collection

**Fig 1:**
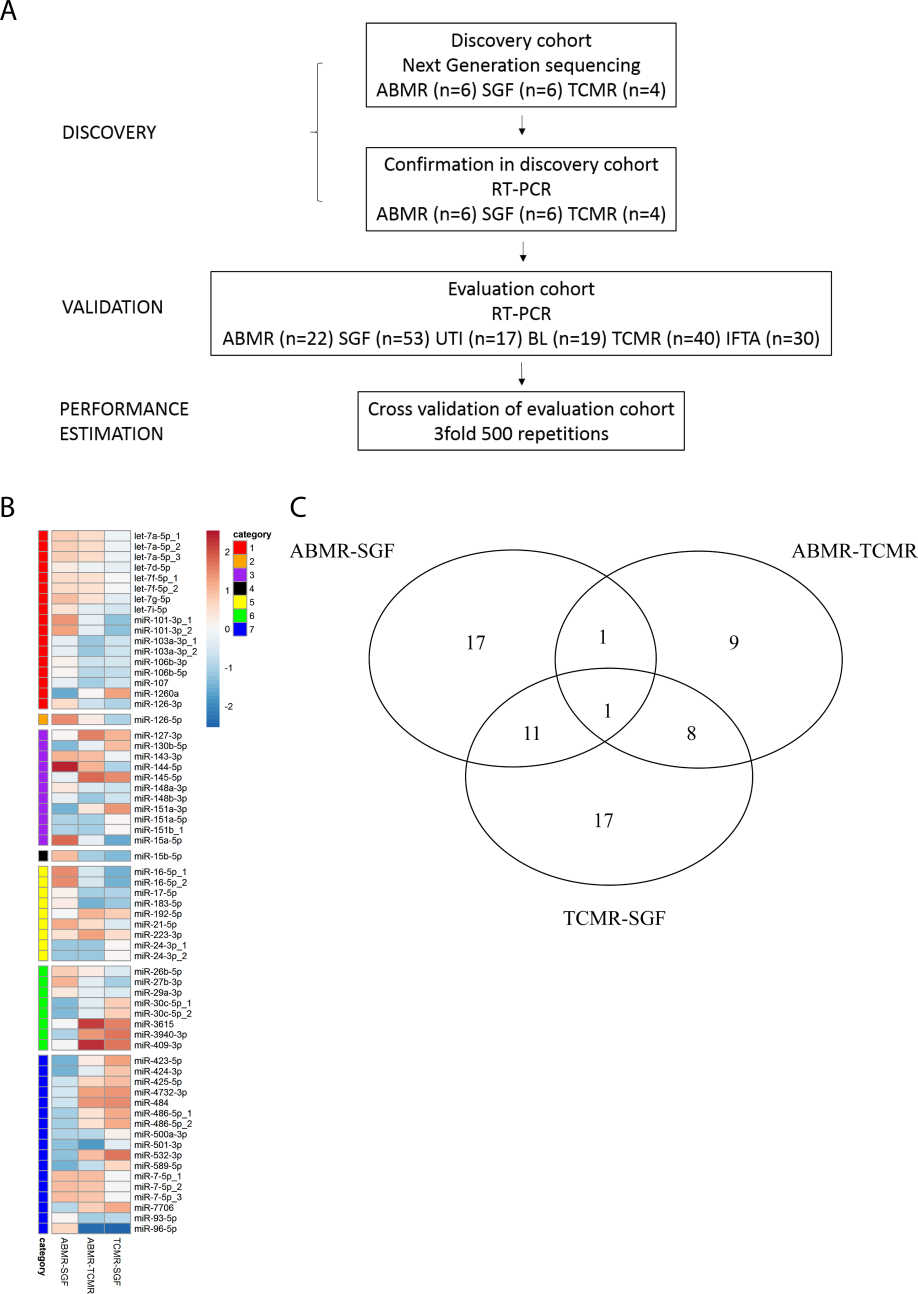
Patient flowchart and analysis of global microRNA expression in patients with ABMR, SGF and TCMR after KTx. Fig 1A Patient flowchart Fig 1B Heatmap showing 64 differentially expressed microRNAs, which distinguish between patients with ABMR, SGF and TCMR. Regulated microRNAs were sorted by categories according to the Venn-diagram (Fig 1C), and displayed as fold-induction (red) and fold-repression (blue); categories 1: ABMR-SGF; 2: ABMR-SGF AND ABMR-TCMR; 3: ABMR-SGF AND TCMR-SGF; 4: regulated in all comparisons; 5: ABMR-TCMR; 6: ABMR-TCMR AND TCMR-SGF; 7: TCMR-SGF Fig 1C Venn-diagram displaying the number of differentially regulated microRNAs between patients with ABMR, SGF and TCMR based on high-throughput Sequencing data. Genes were considered differentially expressed when having a p-value<0.05, and an absolute fold change ≥1.5. Intersections of circles are genes that are significantly regulated in 2 or 3 comparisons.

Adult renal transplant recipients were recruited from the Department of Nephrology, Campus Mitte, Universitätsmedizin Charité, Germany and provided written informed consent. The study was approved by the local ethical committee (Ethikkommission der Charité-Universitaetsmedizin Berlin). 111 blood samples were collected in PAXgene blood RNA tubes (PreAnalytiX, Becton Dickinson, Heidelberg, Germany) from 111 patients at the time of biopsy, 53 samples from 53 control patients with stable graft function and 17 samples from 17 control patients with UTI. Histology was classified according to the Banff09/13 criteria and carried out by two experienced nephropathologists in blinded fashion. 19 patients were diagnosed with BL rejection, 40 patients with TCMR, 22 with ABMR and 30 patients with IFTA. Patients were eligible for inclusion in the SGF control group when a lasting functioning graft could be observed. The 53 SGF patients were monitored in the outpatient clinic for a mean of 21 times during the 12 months after sample collection. Two patients required biopsies during that time without rejection or IFTA diagnosis and two patients died to causes non-related to the transplantation or graft. A flowchart of patient sample use is depicted in Fig 1A, patient demographics are summarized in S1 and S2 Tables.

Total RNA (tRNA) was isolated from blood cells contained in PAXgene Blood RNA Tubes with the PAXgene blood miRNA Kit (PreAnalytix, Qiagen, Hilden, Germany) according to the manufacturers instruction. Total RNA concentration and quality of each sample were determined with the NanoDrop ND-Lite (peqlab, Erlangen, Germany) and Qubit fluorometer (ThermoFisher Scientific, Darmstadt, Germany). Dependent on quantity and quality of RNA, six samples from patients with ABMR, six samples from patients with SGF and four samples from patients with TCMR were chosen for high-throughput sequencing.

### High-throughput sequencing and data analysis

The “TruSeq^®^ small RNA library Prep” kit (Illumina, San Diego, CA, USA) was used to prepare cDNA libraries for small RNA sequencing from a maximum of 1μg of total RNA isolated from the whole blood of patients with KTx according to manufacturer’s instruction. RNA quality was assessed on a bioanalyzer using the RNA 6000 Pico Kit (Agilent Technologies, Waldbronn, Germany) and only RNA with a RIN >8 was considered for further processing. After quality control (High sensitivity DNA Kit, Agilent Technologies, Waldbronn, Germany) the resulting cDNA libraries were purified by gel-electrophoresis for the small RNA containing cDNA fraction at ~150 bp. The final small RNA cDNA libraries were quality checked (High sensitivity DNA Kit, Agilent Technologies, Waldbronn, Germany) and quantified (Qubit dsDNA HS Assay Kit, Invitrogen, Darmstadt, Germany). The final libraries were single-end (50 bp) sequenced on a HiSeq2500 Illumina Next Generation Sequencing Device (Illumina, San Diego, CA, USA). MicroRNAs were identified and quantified using miRDeep2.0.0.8 [16]. Differential expression analyses were performed using DESeq2 [17]. MicroRNAs with p-values below 0.05 and an absolute fold change ≥1.5 were considered significantly differentially expressed. Principal component analyses were based on rlog-normalized reads.

### Quantification of microRNAs

Total RNA was isolated as described above and subsequently, three ng tRNA were reverse transcribed with the Applied Biosystems™ TaqMan™ Advanced miRNA cDNA Synthesis Kit (ThermoFisher Scientific, Germany). TaqMan RT-PCR was performed in duplicate with Applied Biosystems™ TaqMan™ Advanced miRNA Assays (ThermoFisher Scientific, Germany) and TaqMan®Universal MasterMixII (ThermoFisher Scientific, Germany) for candidate markers and endogenous control. Expression of hsa-miR-186-5p was used for normalization as recommended by the manufacturer (ThermoFisher Scientific, Germany) given by the formula (2^-Δ*C*^_t_).

### Statistical analysis RT-PCR

Analysis of variance for continuously distributed parameters and logistic regression analysis for categorical parameters were applied to analyse differences in the distribution between the patient groups, respectively. Differences between the single groups were tested by Post-hoc tests.

For validation of seven candidate microRNAs that were picked from the sequencing data, a nonparametric Mann-Whitney U test was performed to compare ABMR (n = 6), SGF (n = 6) and TCMR (n = 4) patient groups with each other for their miR-223-3p, miR-127-3p, miR-192-5p, miR-409-3p, miR-3615, miR-424-3p and miR-145-5p expression.

To compare the expression data for validated candidates miR-223-3p, miR-409-3p, miR-424-3p and miR-145-5p in a large patient cohort and to correct for multiple testing, a nonparametric 1-way analysis of variance was performed (Kruskal-Wallis test). If samples from the 6 different patient groups did not originate from the same distribution and therefore a significant p-value of below 0.05 was observed, the two-stage step-up method of Benjamini, Krieger and Yekutieli [18, 19], an improved adaptive modification of the Benjamini and Hochberg method [20] with more power, was applied to compare 2 patient groups with each other. Values of the corrected p value (q) smaller than 0.05 were considered statistically significant. The diagnostic value for the classification of IFTA versus SGF as well as versus all other patient groups combined by miR-145-5p expression was evaluated by receiver operating characteristics (ROC) analysis. The optimal cutoff value was defined by the maximal Youden’s index. The 3 fold cross-validation was performed based on a logistic regression model with 500 repetitions and same prevalence in the training and test sets. The analyses were performed using the ROCR and OptimalCutpoints packages in R [21, 22].

## Results

### Identification of candidate microRNAs with high throughput sequencing

High Throughput Sequencing was performed with samples from patients with biopsy-proven ABMR and TCMR and patients with SGF. Sequence analysis showed 301 detectable microRNAs (S3 Table) including 64 microRNAs that were differentially expressed between the three patient cohorts (Fig 1B). The expression of 30 microRNAs distinguished ABMR from control patients, 19 differentiated ABMR from TCMR. Two of these microRNAs were able to discriminate ABMR from controls and from TCMR. 37 microRNAs were differentially expressed between control patients and TCMR, whereas 12 of these differentiated controls from ABMR and from TCMR. Nine specific microRNAs distinguished TCMR from ABMR and controls. Ultimately the expression of 1 microRNA discriminated all three patient groups from each other (Fig 1C).

**Fig 2:**
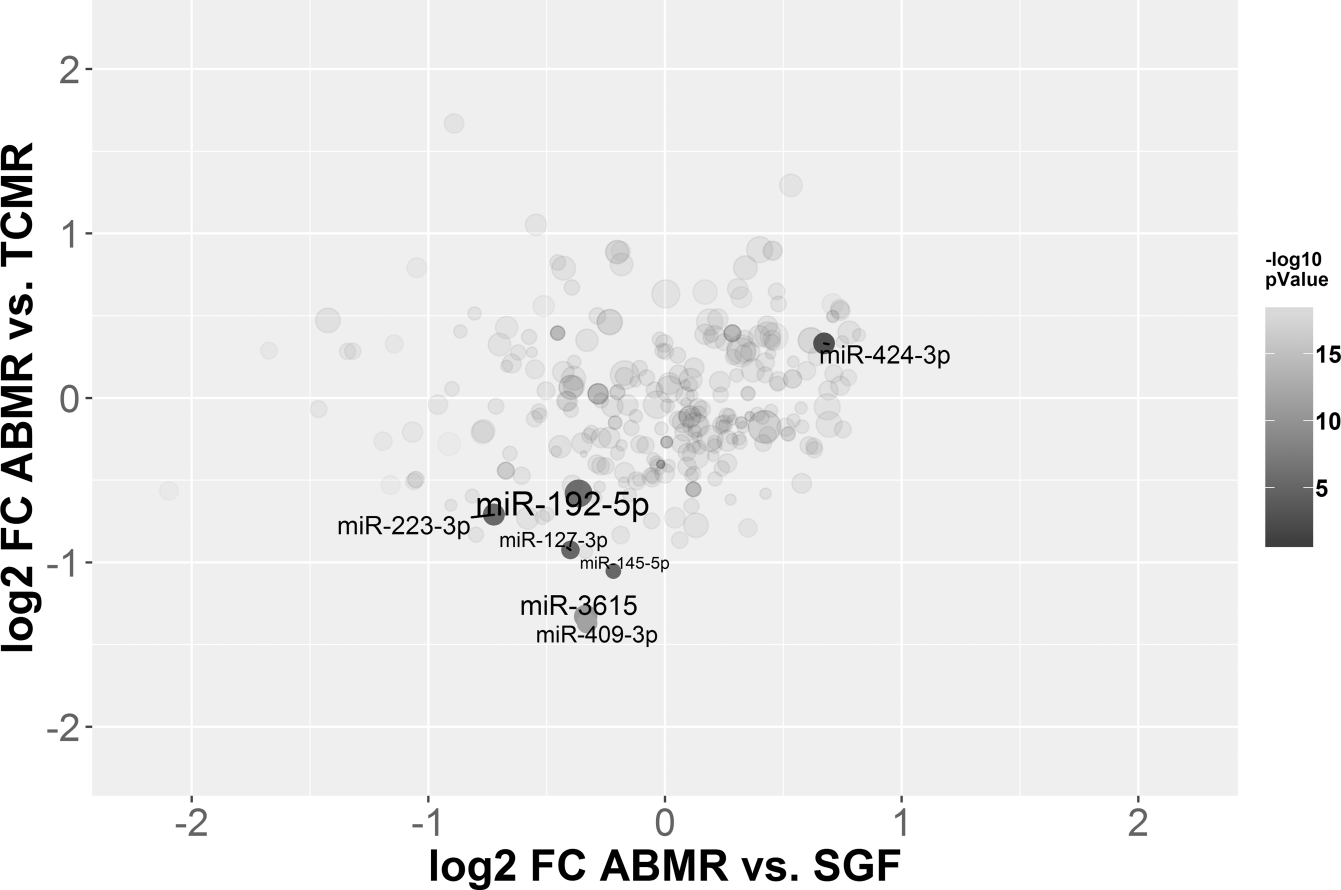
Log2 fold changes of the comparisons ABMR vs. SGF plotted against the log2 fold changes of ABMR vs. TCMR. The size of the microRNA dot is based on the log10 transformed mean expression, and the intensity of colour is based on the -log10 transformed lower p-value of the two comparisons. Annotated are the microRNAs which were further analyzed.

Focusing on the potential of microRNAs as indicators of ABMR, seven candidates were picked according to their ability to distinguish ABMR from TCMR or stable graft function or both (Fig 2). Candidates were also picked due to their expression and the variability in their expression within the single patients cohorts. Particularly, those differentially expressed microRNA which previously appeared to selectively distinguish between TCMR and other pathologies, including miR-15a, miR-15b, miR-16, miR-103, miR-106 and miR-107 [23] were not further evaluated.

### Validation of candidate microRNAs with RT-PCR

**Fig 3:**
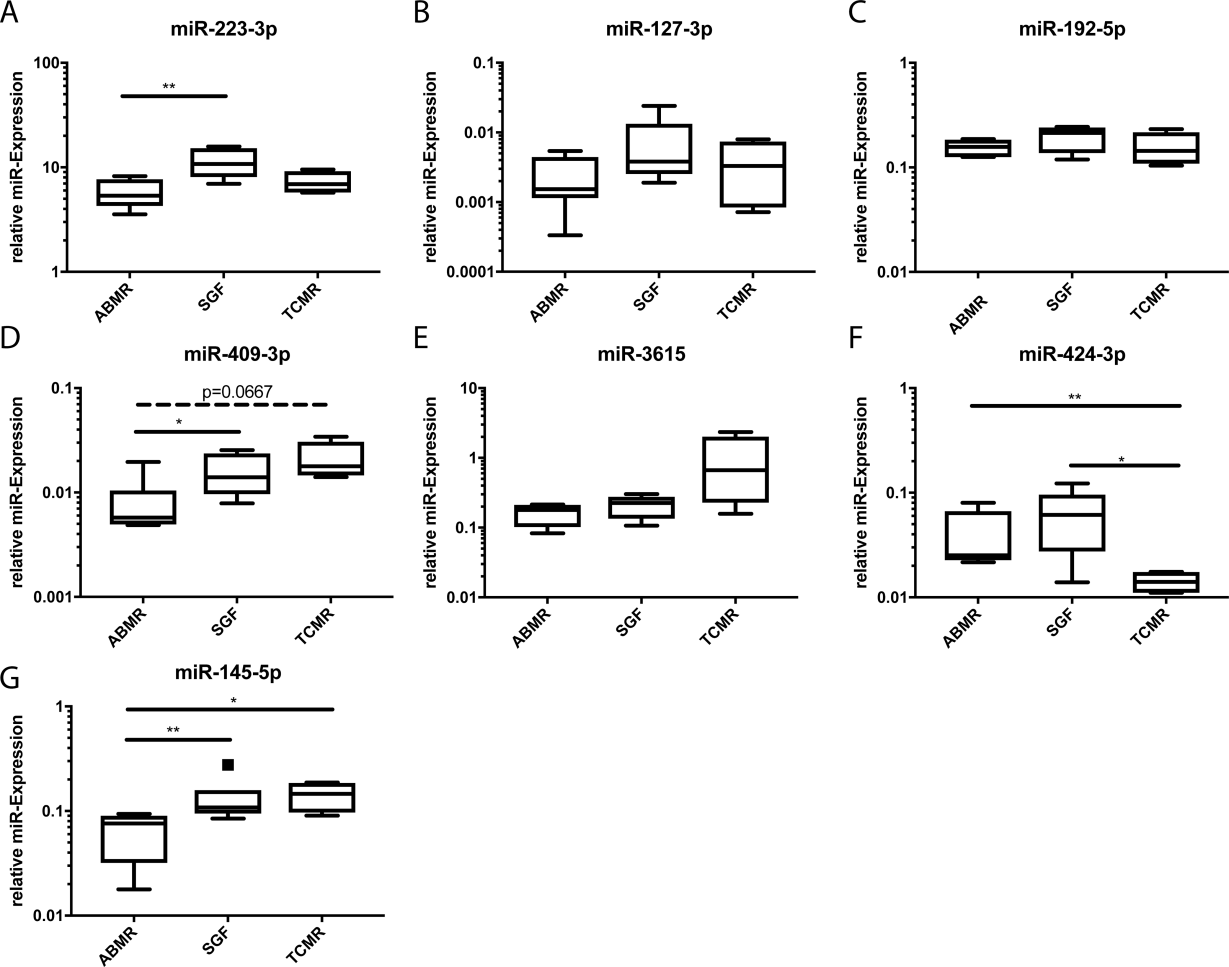
Validation of high-throughput sequencing data with RT-PCR in the same samples. Verification of differential expression of candidate microRNAs in blood cells of patients with ABMR (n = 6), SGF (n = 6) and TCMR (n = 4). Depicted is the relative expression of each microRNA. *p<0.05; **p<0.01.

Candidates from the high-throughput Sequencing experiments were analysed in the same samples by RT-PCR for validation. Hsa-miR-186-5p was used for normalization with robust Cts (24.1 median, 1.85 SD). MiR-223-3p as the only marker distinguishing ABMR from TCMR and SGF in the sequencing experiments could be validated as indicator for ABMR compared to SGF (p = 0.0087) (Fig 3A). MiR-127-3p and miR-192-5p sequences were detected at significantly lower levels when comparing ABMR patients with TCMR patients, which could not be validated with RT-PCR (Fig 3B and 3C). The sequencing data of miR-409-3p and miR-3615 discriminated between TCMR and ABMR as well as between TCMR and SGF. For miR-409-3p the validation analysis discovered an additional significant difference between ABMR and SGF (p = 0.026), no difference between TCMR and SGF but a trend towards difference between ABMR and TCMR (p = 0.0667) were observed (Fig 3D). No significant differences resulted from the validation data for miR-3615 (Fig 3E). While showing a significant difference in the sequencing analysis between ABMR and SGF, miR-424-3p displayed significantly down-regulated expression levels in TCMR compared to ABMR (p = 0.0095) and SGF (0.0381) (Fig 3F). The sequencing results for miR-145-5p were validated by RT-PCR for the significant difference between ABMR and TCMR (p = 0.019), an additional significant difference in expression levels was observed for ABMR versus SGF (p = 0.0087) (Fig 3G).

### Regulation of candidate microRNAs after KTx

The potential of candidates miR-127-3p, miR-192-5p and miR-3615 as indicators of ABMR after kidney transplantation could not be validated (see Fig 3), therefore these markers were excluded from further analyses. The remaining candidates were measured in a large cohort of patients (n = 181) with SGF and different pathologies including infections (UTI), different types of rejection (ABMR, TCMR, BL) and also IFTA.

**Fig 4:**
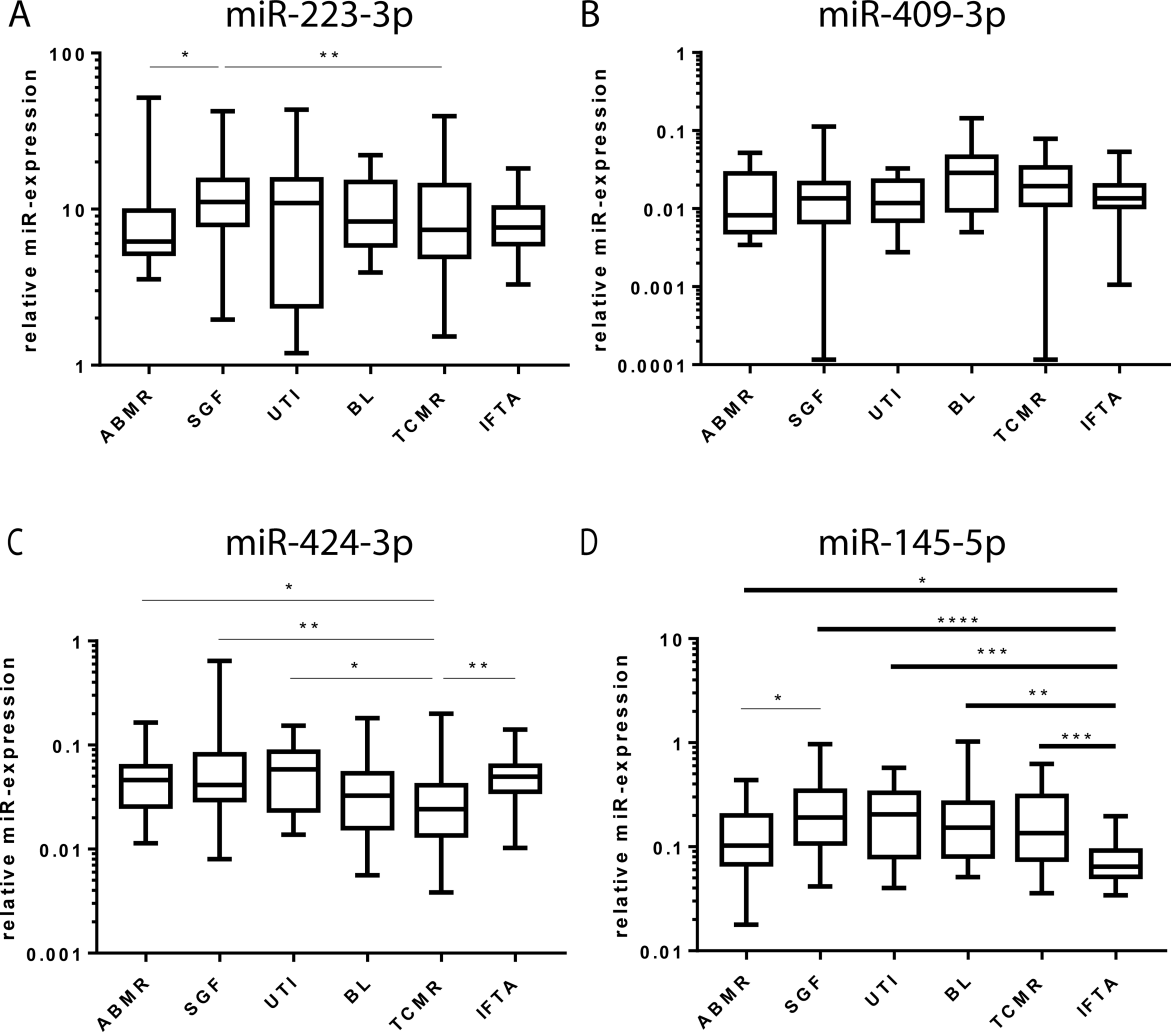
MicroRNA expression in blood cells after KTx. After performing a non-parametric ANOVA (Kruskal-Wallis test) to correct for multiple testing, multiple comparisons with the two-stage linear step-up procedure of Benjamini, Krieger and Yekutieli were applied. The box-Whisker-Plots (Tukey) show the median expression of miR-223-3p (A), miR-409-3p (B), miR-424-3p (C) and miR-145-5p (D) in blood cells from patients with ABMR, SGF, UTI, BL, TCMR and IFTA. * corrected p-value <0.05; ** corrected p-value <0.01; *** corrected p-value <0.001; **** corrected p-value <0.0001.

A non-parametric Kruskal-Wallis Test was performed for all patient groups and every microRNA to assess significant differences. The test revealed significant distinctions between the groups for miR-223-3p (p = 0.0163), miR-409-3p (p = 0.0274), miR-424-3p (p = 0.0009) and highly significant distinctions for miR-145-5p (p<0.0001). Subsequently, a two-stage step-up test of Benjamini, Krieger and Yekutieli [18] between any two groups was performed. For miR-223-3p only two significant expression levels became apparent, namely between ABMR and SGF (q = 0.031) and between TCMR and SGF (q = 0.0486) (Fig 4A). For miR-409-3p no significant differences between the groups were discovered (Fig 4B). The expression levels of miR-424-3p were significantly downregulated when comparing TCMR to ABMR (q = 0.0445), SGF (q = 0.0039), UTI (q = 0.0125) and IFTA (q = 0.0027) but not BL and not when comparing any other groups with each other (Fig 4C). MiR-145-5p expression levels were significantly different when comparing ABMR with SGF (q = 0.0171). Additional results were discovered regarding the highly significant differences in miR-145-5p expression when comparing IFTA to ABMR (q = 0.0171), SGF (q<0.0001), UTI (q = 0.0007), BL (q = 0.0013) and TCMR (q = 0.0004) (Fig 4D).

### Diagnostic value of candidate miR-145-5p for IFTA after KTx

**Fig 5:**
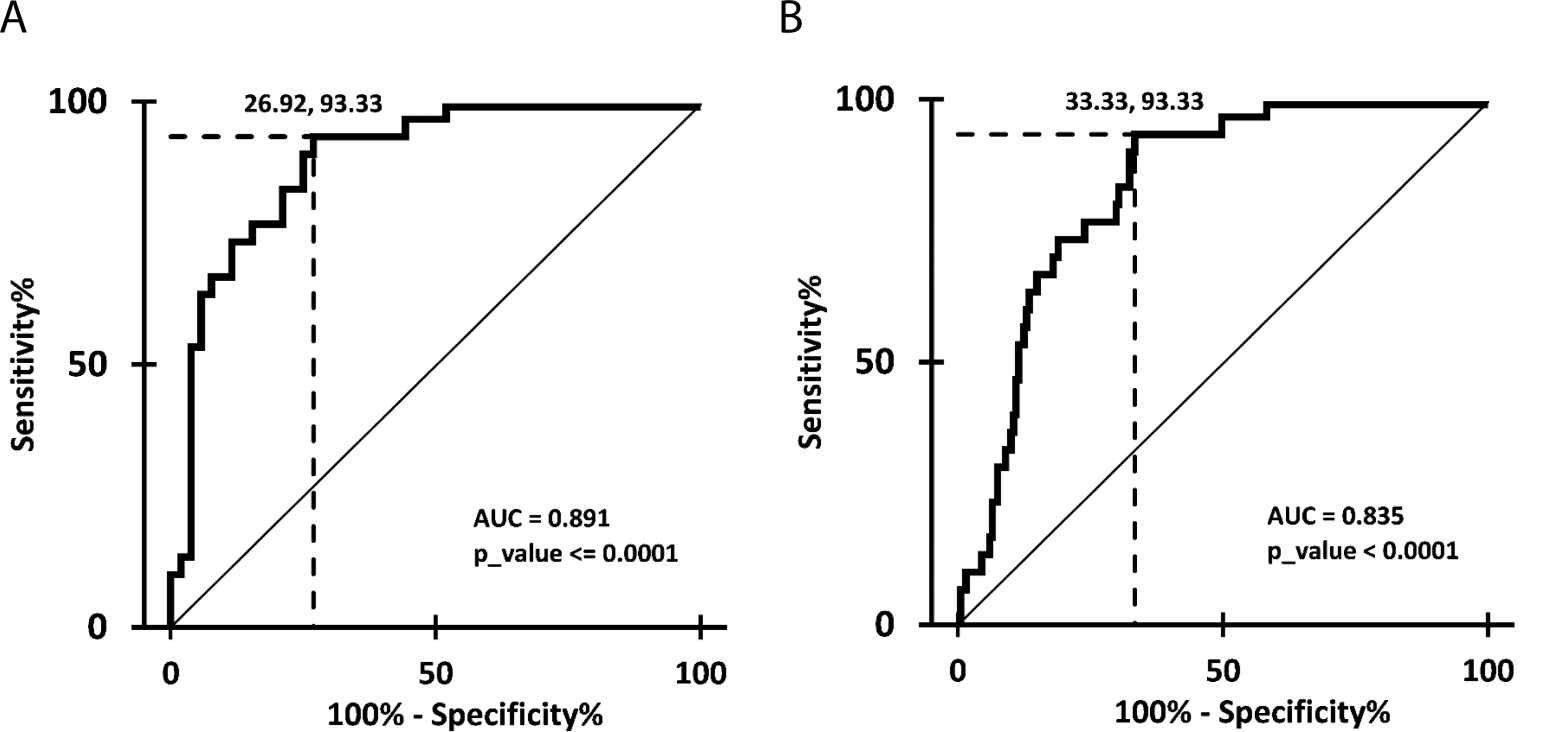
Diagnostic properties of miR-145-5p expression for IFTA after KTx. ROC curves for miR-145-5p expression levels in blood cells from patients with histologically diagnosed IFTA versus patients with SGF (A) and SGF, ABMR, UTI, BL and TCMR combined (B) demonstrate sensitivities and specificities. Dashed lines indicate the optimal cutoff, as judged by the Youden index.

ROC analysis of miR-145-5p expression in IFTA patients versus patients with SGF resulted in an AUC of 0.891 (p-value <0.0001). At the optimal cut-off of 0.111, as judged by the Youden index, patients showed a sensitivity of 93% and a specificity of 73% (Fig 5A, Table 1). In comparison, ROC analysis of IFTA and all other patients combined revealed an AUC of 0.835 (p-value <0.0001), a sensitivity of 93% and specificity of 67% at a similar optimal cut-off of 0.111 (Fig 5B, Table 1). A 3-fold cross validation with 500 repeats verified that miR-145-5p would indeed perform accurately in practice. The summary of diagnostic properties for our candidate miR-145-5p after cross validation is presented in Table 1.

**Table 1:** Diagnostic performance of miR-145-5p expression for IFTA.

**A**	**cuttoff**	**AUC (95% CI)**	**Youden Index**	Sensitivity* **(95% CI)**	Specificity* **(95% CI)**	LR+* **(95% CI)**	LR-* **(95% CI)**
**IFTA vs SGF**	0.111	0.891 (0.821–0.961)	0.664	0.933 (0.779–0.992)	0.731 (0.595–0.844)	3.467 (2.193–5.480)	0.091 (0.024–0.353)
**IFTA vs all other**	0.111	0.835 (0.773–0.896)	0.600	0.933 (0.779–0.992)	0.667 (0.597–0.731)	2.800 (2.252–3.481)	0.100 (0.026–0.383)
**B**	**cuttoff**	**AUC (95% CI)**	**Youden Index**	Sensitivity* **(95% CI)**	Specificity* **(95% CI)**	LR+* **(95% CI)**	LR-* **(95% CI)**
**IFTA vs SGF**		0.887 (0.884–0.890)		0.902 (0.897–0.906)	0.707 (0.701–0.712)	3.074 (3.014–3.135)	0.139 (0.132–0.146)
**IFTA vs all other**		0.832 (0.829–0.835)		0.918 (0.913–0.922)	0.646 (0.643–0.649)	2.595 (2.570–2.620)	0.127 (0.120–0.134)

ROC analysis of data from patients with IFTA versus SGF and versus the collective data of all other groups (SGF, UTI, BL, TCMR, ABMR) was performed to determine optimal thresholds for the classification of blood samples and also to estimate the diagnostic value of each miR-145-5p level (A). The performance of the classification was cross-validated based on a univariate logistic regression model in a 3 fold and 500 repeats setting (B). AUC…area under the curve; CI…confidence interval; LR…likelihood ratio

*… at optimal Youden index

## Discussion

Insights into the regulation of microRNAs in blood cells after KTx cannot only lead to the discovery of potential biomarkers but also to a deeper understanding of the diverse molecular mechanisms involved in completely differential pathologies and their progression. Especially ABMR has been identified as the leading cause of graft dysfunction and loss, since precise diagnostic approaches and curative therapy strategies are lacking. Our goal was to identify microRNA candidates that are regulated during events of ABMR via high throughput sequencing as initial screening step. We further aimed to evaluate their capacity of distinguishing ABMR not only from SGF, but also from conditions requiring aberrant therapeutical consequences including infections, BL rejections, TCMR and IFTA in a large patient cohort.

The analysis of sequencing data revealed only one microRNA, whose regulation in blood cells was supposedly differential between patients with SGF, ABMR and TCMR. Surprisingly, this miR-15b-5p had recently been discovered by our group as a highly specific marker for T-cell mediated vascular rejection (TCMVR). In this study a significantly different miR-15b-5p expression was observed when comparing patients with ABMR and TCMVR, but not when comparing ABMR patients with SGF patients. [23]. The list of 64 significantly regulated microRNAs included miR-15a, miR-16 and miR-107, which had also been analysed in the study mentioned above. We therefore focused on other candidates that would either be discriminative between ABMR and TCMR, ABMR and SGF or both.

The highly promising candidate miR-223-3p has so far been introduced as potential regulator in synovial sarcoma and gastric cancer [24, 25] It has also been studied in human and mouse fibroblast cell lines in the context of rheumatoid arthritis [26] and in biopsy material of patients with Crohn`s Disease [27]. In the sequencing experiments miR-223-3p levels were significantly different between ABMR and SGF as well as TCMR. When analysing this candidate in the six patient groups, only the expression difference between ABMR and SGF could be confirmed. This finding does not qualify miR-223-3p as a specific marker for ABMR due to the absence of significant expression differences to the other pathologies and therefore excludes this microRNA as a selective biomarker for ABMR. This consequence applies to the candidates miR-409-3p and miR-424-3p, which both seem to play important roles in diseases like oesophageal adenocarcinoma, lung carcinoids, osteosarcoma, breast cancer [28–32]. MiR-409-3p measurement in the large patient cohort did not display any significant differences between ABMR and the other groups and miR-424-3p detection only indicated significant expression level differences between ABMR and TCMR. Interestingly, miR-424-3p expression levels in TCMR were not only lower compared to ABMR, but also when comparing them to SGF, UTI and IFTA. A significant difference was not observed between TCMR and BL, which would be essential for a selective and clinically useful biomarker for TCMR. Nevertheless, the finding regarding miR-424-3p expression in TCMR after KTx would be suitable for further biomarker and also mechanistic studies.

MiR-145-5p is abundant in immature blood cells including hematopoietic stem/progenitor cells. It is a member of the miR-143/145 cluster with supposedly tumor affecting functions. MiR-143/145 are highly expressed in smooth muscle cells, and in the vascular wall of normal healthy blood vessels. Interestingly, this cluster has been studied more intensively than other candidates in regard to regulation and its tumorigenesis affecting function [33–35], including in the context of renal pathologies. MiR-143/145 has recently been reported to be involved in hydronephrosis in mice [36]. In renal cell carcinoma cell lines miR-145-5p is dysregulated and several targets, including e2F-associated phosphoprotein (EAPP), Heparan Sulfate 6-O-Sulfotransferase 2 (HS6ST2), Lysyl Oxidase (LOX), Transforming Growth Factor beta-2 (TGFB2) and Vaccinia-related Kinase 2 (VRK2), were confirmed [37]. Our observation of a regulated miR-145-5p expression between ABMR and TCMR in blood cells after KTx could not be confirmed in a large patient cohort. Significantly reduced expression levels were found when comparing SGF with ABMR. This finding does not illustrate an actual diagnostic value since the different types of rejection cannot be distinguished from each other. Unexpectedly the miR-145-5p expression levels in the IFTA group were significantly lower compared to all other pathologies and SGF. The ROC analysis of miR-145-5p expression in IFTA versus SGF and also versus all other groups combined showed a high diagnostic value with AUCs of 0.891 and 0.835, resp., which was confirmed by cross validation. Nevertheless, we highly recommend a validation of this data in an independent patient cohort with similar or extended control groups. Especially the data regarding the UTI group needs to be verified in a cohort with stable UTI patients that received protocol biopsies to exclude patients that might develop IFTA. Additionally, it is important to mention that the time post KTx in IFTA patients was longer then in the patients with SGF, which allows the assumption of absent IFTA in the latter group, but still suggests the integration of matched groups in following studies.

IFTA is a descriptive term that has replaced the non-specific morphologic diagnosis “Chronic Allograft Nephropathy” since the Banff 2005 meeting [38]. It can be induced by diverse immune and non-immune factors and is only diagnosed when the actual etiology of tubulointerstitial fibrosis and tubular atrophy is not clearly determinable. Of note, miR-145-5p expression is downregulated in ABMR and IFTA cohorts when compared to the other groups. Since the Banff classification for ABMR includes the occurrence of fibrosis, a similar regulation of miR-145-5p expression in ABMR and IFTA might be explainable. Interestingly, miR-145 has recently been linked to fibrosis by overexpressing it in chondrocyte cell lines causing a significant inhibition of proliferation and fibrosis. The same observation was made when knocking down the assumed target tumor necrosis factor receptor superfamily, member 11b, in vitro [39]. In contrast, miR-145 -/- mice develop less severe lung fibrosis than wild-type mice in a pulmonary fibrosis model [40]. We therefore suggest in-depth studies to identify the main cell source of miR-145-5p in blood cells and we hypothesize that this particular cell population might be recruited to the graft contributing to the mechanisms that lead to fibrosis and potentially also to the fibrotic changes contributing to ABMR. This might point to our observation of significantly reduced miR-145-5p expression levels in blood cells of IFTA patients compared to patients with TCMR, BL, UTI and SGF. In this context we would highly recommend the realisation of a new study with high-throughput sequencing experiments putting the focus on IFTA in correlation to ABMR after KTx to identify additional markers that might be involved in the processes described above. Most microRNA studies focus on biomarker search rather than on target gene identification, regulation and the functional context. It is known that miR-145 might control hundreds of target genes. Toll–interleukin-1 receptor domain–containing adaptor protein (TIRAP) has been studied in detail as presumable target. The loss of miR-145 and miR-146 in hematopoietic stem/progenitor cells and the resulting increase in the expression of their targets TIRAP and TNF receptor associated factor 6 (TRAF6), may lead to the activation of innate immune signaling through TRAF6-mediated immune signaling [41]. The Toll-like receptor 4 associated adapter molecule TIRAP is involved in the signaling pathways leading to the activation of NF-kappa-B, MAPK1, MAPK3 and JNK. It has been suggested, that TLR4 enhances hepatic fibrosis through several mechanisms [42] and that TLR4 plays distinct roles in the pathogenesis of renal fibrosis [43, 44]. Our data leads us to the hypothesis that miR-145-5p might be involved in the TLR-mediated mechanisms leading to fibrosis -especially inflammatory IFTA- in renal grafts. This assumption requires further intense functional investigations that are beyond the scope of this manuscript, including the identification of cell types producing miR-145-5p and the elucidation of mechanisms leading to the down-regulation of expression levels in peripheral blood cells. Interestingly, in a substudy calcineurin inhibitor nephrotoxicity proved to be uninvolved in the specific down-regulation of miR-145-5p expression in IFTA-patients. In this context the multifaceted etiologies leading to IFTA after KTx pose a major challenge and have to be considered in further studies.

## Supporting information

S1 Table Patient demographics. Scr…serum creatinine; m…male; f…female; l…living; nl…non-living; r…related; ur…unrelated. CNI…calcineurin inhibitor; PI…Proliferation inhibitor; St…steroids; mTORi…mTOR inhibitor; Bela…Belatacept.
(DOCX)

S2 Table Statistical evaluation of Patient parameters. Analysis of variance for continuously distributed parameters and logistic regression analysis for categorical parameters were applied to analyse differences in the distribution between the patient groups, respectively. Differences between the single groups were tested by Post-hoc tests. For the statistical analysis regarding the parameters “days post Tx”, “creatinine in the serum” and “age of the recipients” we performed a non-parametric ANOVA (Kruskal-Wallis test). Whenever the ANOVA analysis turned to be significant (p<0.05) a Dunn’s multiple comparison test between single groups was performed. (-) statistics not possible, *** corrected p-value <0.001.
(DOCX)

S3 Table miR Deep extracted miRNome. (XLSX)
