# Supplementary material for: MicroRNA regulation in blood cells of renal transplanted patients with interstitial fibrosis/tubular atrophy and antibody-mediated rejection
Source: PLoS One. 2018 Aug 13;13(8):e0201925. doi: 10.1371/journal.pone.0201925 (PMC6089438; doi:10.1371/journal.pone.0201925)
Supplement: S1 Table — Scr…serum creatinine; m…male; f…female; l…living; nl…non-living; r…related; ur…unrelated. CNI…calcineurin inhibitor; PI…Proliferation inhibitor; St…steroids; mTORi…mTOR inhibitor; Bela…Belatacept. (DOCX) [file pone.0201925.s001.docx]

|  | **ABMR** | **SGF** | **UTI** | **BL** | **TCMR** | **IFTA** |
| --- | --- | --- | --- | --- | --- | --- |
| **age recipient**  **(mean,SD)** | 45.1  13.1 | 51.0  15.8 | 52.0  17.0 | 48.5  14.9 | 51.7  14.7 | 57.2  14.9 |
| **sex recipient** | 18 m  4 f | 33 m  20 f | 2 m  15 f | 12 m  7 f | 32 m  8 f | 17 m  13 f |
| **days post Tx**  **(mean,SD)** | 2714  2465 | 33  50 | 2638  2634 | 690  973 | 301  619 | 2528  2575 |
| **SCr in mg/dL**  **(mean,SD)** | 3.5  1.8 | 1.5  0.6 | 2.1  1.2 | 3.5  2.0 | 6.5  3.8 | 3.7  2.4 |
| **previous KTx** | 18 0  3 1  1 2 | 52 0  1 1  0 2 | 10 0  7 1  0 2 | 18 0  1 1  0 2 | 34 0  4 1  2 0 | 26 0  3 1  1 2 |
| **sex donor** | 9 m  13 f  0 uk | 28 m  25 f  0 uk | 9 m  6 f  2 uk | 8 m  11 f  0 uk | 23 m  17 f  0 uk | 15 m  13 f  2 uk |
| **(non)living donor** | 10 l  12 nl  0 uk | 24 l  29 nl  0 uk | 6 l  9 nl  2 uk | 10 l  9 nl  0 uk | 9 l  31 nl  0 uk | 4 l  24 nl  2 uk |
| **(un)related donor** | 8 r  2 ur  0 uk | 12 r  12 ur  0 uk | 4 r  2 ur  2 uk | 7 r  3 ur  0 uk | 5 r  4 nr  0 uk | 2 r  2 ur  0 uk |
| **BKV nephropathy** | 21 negative  0 positive  1 unknown | - | - | 19 negative  0 positive  0 unknown | 40 negative  0 positive  0 unknown | 30 negative  0 positive  0 unknown |
| **Immunosuppressive therapy** |  |  |  |  |  |  |
| **CNI/PI/St** | 9 | 52 | 5 | 12 | 34 | 14 |
| **CNI/St** | 1 | 0 | 1 | 0 | 2 | 4 |
| **CNI/PI** | 8 | 0 | 7 | 4 | 0 | 7 |
| **CNI** | 0 | 0 | 2 | 0 | 0 | 1 |
| **PI/St** | 4 | 0 | 1 | 0 | 1 | 3 |
| **St** | 0 | 0 | 0 | 0 | 0 | 1 |
| **St/mTORi** | 0 | 0 | 0 | 0 | 1 | 0 |
| **PI/mTORi** | 0 | 0 | 0 | 3 | 1 | 0 |
| **St/Bela** | 0 | 0 | 1 | 0 | 0 | 0 |
| **PI/ St/Bela** | 0 | 1 | 0 | 0 | 1 | 0 |

**S1 Table Patient demographics and immunosuppressive therapy**

Scr…serum creatinine; m…male; f…female; l…living; nl…non-living; r…related; ur…unrelated

CNI…calcineurin inhibitor; PI…Proliferation inhibitor; St…steroids; mTORi…mTOR inhibitor; Bela…Belatacept
