# Supplementary material for: MicroRNA regulation in blood cells of renal transplanted patients with interstitial fibrosis/tubular atrophy and antibody-mediated rejection
Source: PLoS One. 2018 Aug 13;13(8):e0201925. doi: 10.1371/journal.pone.0201925 (PMC6089438; doi:10.1371/journal.pone.0201925)
Supplement: S2 Table — Analysis of variance for continuously distributed parameters and logistic regression analysis for categorical parameters were applied to analyse differences in the distribution between the patient groups, respectively. Differences between the single groups were tested by Post-hoc tests. For the statistical analysis regarding the parameters “days post Tx”, “creatinine in the serum” and “age of the recipients” we performed a non-parametric ANOVA (Kruskal-Wallis test). Whenever the ANOVA analysis turned to be significant (p<0.05) a Dunn’s multiple comparison test between single groups was performed. (-) statistics not possible, *** corrected p-value <0.001. (DOCX) [file pone.0201925.s002.docx]

|  | **ABMR vs. SGF** | **ABMR vs. UTI** | **ABMR vs. BL** | **ABMR vs. TCMR** | **ABMR vs. IFTA** | **SGF vs. UTI** | **SGF vs. BL** | **SGF vs. TCMR** | **SGF vs. IFTA** | **UTI vs. BL** | **UTI vs. TCMR** | **UTI vs. IFTA** | **BL vs. TCMR** | **BL vs. IFTA** | **TCMR vs. IFTA** |
| --- | --- | --- | --- | --- | --- | --- | --- | --- | --- | --- | --- | --- | --- | --- | --- |
| **sex recipient** | 0.106 | <0.001 | 0.186 | 0.862 | 0.063 | 0.002 | 0.945 | 0.069 | 0.617 | 0.004 | <0.001 | 0.006 | 0.171 | 0.653 | 0.039 |
| **previous KTx** | 0.069 | 0.071 | 0.361 | 0.670 | 0.673 | 0.001 | 0.461 | 0.112 | 0.129 | 0.026 | 0.014 | 0.021 | 0.516 | 0.541 | 0.981 |
| **sex donor** | 0.349 | 0.257 | 0.938 | 0.214 | 0.375 | 0.623 | 0.424 | 0.654 | 0.949 | 0.303 | 0.867 | 0.686 | 0.271 | 0.441 | 0.748 |
| **(non)living donor** | 0.989 | 0.742 | 0.647 | 0.065 | 0.020 | 0.716 | 0.583 | 0.025 | 0.008 | 0.465 | 0.200 | 0.066 | 0.024 | 0.007 | 0.400 |
| **(un)related donor** | 0.119 | 0.554 | 0.608 | 0.262 | 0.277 | 0.469 | 0.291 | 0.776 | 0.999 | 0.889 | 0.668 | 0.600 | 0.517 | 0.486 | 0.853 |
|  |  |  |  |  |  |  |  |  |  |  |  |  |  |  |  |
| **CNI/PI/St** | <0.001 | 0.460 | 0.159 | 0.001 | 0.680 | <0.001 | 0.002 | 0.044 | <0.001 | 0.048 | <0.001 | 0.251 | 0.066 | 0.262 | 0.001 |
| **CNI/St** | - | 0.852 | - | 0.936 | 0.310 | - | - | - | - | - | 0.892 | 0.438 | - | - | 0.235 |
| **CNI/PI** | - | 0.759 | 0.287 | - | 0.309 | - | - | - | - | 0.197 | - | 0.204 | - | 0.852 | - |
| **CNI** | - | - | - | - | - | - | - | - | - | - | - | 0.285 | - | - | - |
| **PI/St** | - | 0.278 | - | 0.061 | 0.399 | - | - | - | - | - | 0.538 | 0.631 | - | - | 0.215 |
| **St** | - | - | - | - | - | - | - | - | - | - | - | - | - | - | - |
| **St/mTORi** | - | - | - | - | - | - | - | - | - | - | - | - | - | - | - |
| **PI/mTORi** | - | - | - | - | - | - | - | - | - | - | - | - | 0.095 | - | - |
| **St/Bela** | - | - | - | - | - | - | - | - | - | - | - | - | - | - | - |
| **PI/ St/Bela** | - | - | - | - | - | - | - | 0.841 | - | - | - | - |  | - | - |
| **Age of the recipient** | n.s. | n.s. | n.s. | n.s. | n.s. | n.s. | n.s. | n.s. | n.s. | n.s. | n.s. | n.s. | n.s. | n.s. | n.s. |
| **Creatinine in the serum** | <0.001 | n.s. | n.s. | n.s. | n.s. | n.s. | <0.001 | <0.001 | <0.001 | n.s. | <0.001 | n.s. | n.s. | n.s. | n.s. |
| **Days after Tx** | <0.001 | n.s. | n.s. | <0.001 | n.s. | <0.001 | n.s. | n.s. | <0.001 | n.s. | <0.001 | n.s. | n.s. | n.s. | <0.001 |
